# Cross-sectional analysis of eating disorder risk and risk correlates in candidates for bariatric surgery from the BariPredict cohort

**DOI:** 10.1038/s41598-025-95614-6

**Published:** 2025-04-01

**Authors:** Dana AlTarrah, Lulwa Al-Abdullah, Mohammed Alhusayan, Dulce Canha, Sulaiman Almazeedi, Ahmad Al-Serri, Maryam Abulhasan, Talia Alsomly, Naif Almutawa, Mohammed Al-Onaizi, Guy Fagherazzi, Fawaz Alzaid

**Affiliations:** 1 Department of Social and Behavioral Science, Faculty of Public Health, Kuwait University, Kuwait City, Kuwait; 2 Dasman Diabetes Institute, Gulf Road intersecting Jassim Al Bahar St., Sharq, Block 3, Dasman, P.O. Box 1180, Kuwait City, Kuwait; 3 Deep Digital Phenotyping Research Unit, Department of Precision Health, Luxembourg Institute of Health, 1A-B, rue Thomas Edison, 1445 Strassen, Luxembourg; 4 Faculty of Science, Technology and Medicine, University of Luxembourg, Esch-sur-Alzette, Luxembourg; 5 Department of Surgery, Jaber Al-Ahmed Hospital, Kuwait City, Kuwait; 6 Department of Pathology, Faculty of Medicine, Kuwait University, Kuwait City, Kuwait; 7 Kuwait Center for Mental Health, Ministry of Health, Kuwait City, Kuwait; 8 Department of Community Medicine and Behavioral Science, Faculty of Medicine, Kuwait University, Kuwait City, Kuwait; 9 Department of Anatomy, Faculty of Medicine, Kuwait University, Kuwait City, Kuwait; 10 INSERM UMR-S1151, CNRS UMR-S8253, Université Paris Cité, Institut Necker Enfants Malades, Paris, France; 11 Present Address: King Faisal Specialist Hospital & Research Centre, Riyadh, Saudi Arabia; 12 Present Address: Department of Cancer Biology, Perelman School of Medicine, University of Pennsylvania, Philadelphia, PA, USA

**Keywords:** Obesity, Obesity

## Abstract

Bariatric surgery is an effective intervention for managing obesity. Persons with obesity are a high-risk population for eating disorders (ED), and these can negatively impact perioperative and long-term outcomes of surgery. We aim to understand prevalence and correlates of ED in preintervention patients, identifying those needing psychological support. Baseline cross-sectional analysis of 275 patients of the BariPredict cohort (NCT06480058), a study to assess predictors of long-term surgery outcomes. Psychological assessments were conducted using SCOFF, KUAS, and BDI tools. Data were analyzed for prevalence of high ED risk and for associations of clinical, biological and demographic factors. Mean age was 38.5 years, mean BMI was 42.3 kg/m², with 62.5% being female. 65.8% of patients had a SCOFF score **≥** 2 indicating high ED risk. Class II obesity (*p* < 0.05), younger age (*p* < 0.01), and higher depression (*p* < 0.01) were associated with ED risk in a logistic regression adjusted for age, obesity class, diabetes, HbA1c, depression and anxiety scores. We report high preintervention prevalence of ED, with a risk profile corresponding to BMI of 35-39.9 Kg/m^2^ in younger adults with concurrent depression. This patient profile should be prioritized for psychological assessment and support to potentially improve outcomes of bariatric surgery.

**Supplementary Information:**

The online version contains supplementary material available at 10.1038/s41598-025-95614-6.

## Introduction

Obesity affects one in eight individuals worldwide, and with rates having more than doubled since 1990, bariatric surgery has become a widely used intervention to lose weight, improve glycemic control, or mitigate risk or severity of type 2 diabetes and cardiovascular disease. Despite other available approaches (lifestyle modification, medical therapy) bariatric surgery is the most effective and durable intervention for individuals living with severe obesity (BMI ≥ 40 kg/m^2^) and BMI between 35.0 and 39.9 kg/m^2^ with co-existence of obesity-related comorbidities^1,2^.

Because candidates for bariatric surgery are prone to disordered eating and other psychological or psychiatric manifestations (anxiety, depression)^3^, a psychiatric evaluation is recommended during individual preoperative assessments^4^. This recommendation comes from the fact that psychiatric conditions can impact short- and long-term post-surgical outcomes, including weight loss, mental health and quality of life^5,6^.

While a consequent number of studies has reported on the development of eating disorders (ED) post-operatively^7–9^, and how these influence weight loss outcomes, fewer recent studies addressed pre-operative screening^10,11^. These studies report that patients seeking bariatric surgery have high rates of psychopathology (anxiety, depressive disorder), and a current diagnosis is associated with greater symptoms of food addiction and night-time eating^11^. Comparable work estimates that up to 47% of individuals with obesity, without weight loss intervention, are high risk for having an ED, as determined by the standardized SCOFF screening tool^12^. Given the scarcity of reports on the subject, especially in the Arabian Gulf region, and the implications that disordered eating has for the choice of intervention and its success, we set up a cohort for screening and prospective follow up from bariatric surgery (BariPredict). BariPredict is a single-center longitudinal cohort study of a population of persons in Kuwait that have been referred for bariatric surgery, data are collected in a hospital setting from a preintervention multidisciplinary team (MDT) screening. Here, we exploit preoperative baseline data from the BariPredict cohort (NCT06480058) to report on the prevalence and correlates of disordered eating risk in bariatric surgery candidates.

We hypothesize that prevalence of disordered eating will be higher in individuals that are candidates for bariatric surgery, than in what has been previously reported for the general population with obesity. In terms of correlates, we hypothesize that other psychiatric conditions, and that degree of obesity and presence of metabolic comorbidities (e.g., diabetes, dyslipidemia) will be associated with higher risk of ED. Our findings will establish a baseline for future follow-up studies to evaluate post-operative outcomes and will indicate which patients may benefit from targeted support for ED.

## Method

### BariPredict cohort

BariPredict cohort is a single-center longitudinal cohort study at Jaber AlAhmad AlSabah hospital (JAAH) an internationally recognized Center of Excellence for Metabolic and Bariatric Surgery by the Surgical Review Cooperation. Patients attending the clinic for bariatric surgery are screened by a MDT. For the 376 participants screened, clinical records were retrieved, and their eligibility and the data completeness were verified.

### Research design and methods

#### Research design

Recruitment was based on consecutive convenience sampling of 376 patients that underwent screening by a MDT in preparation for bariatric surgery at JAAH (preparticipation procedures in supplementary methods). Of the 376 patients, 275 had complete baseline data and served as the subgroup on which cross-sectional analysis was performed (Fig. S1 and supplementary methods for eligibility and missing data). The analysis investigated prevalence of ED risk and association of multiple factors (psychological, physical).

#### Inclusion and exclusion criteria

##### Inclusion criteria

obesity (BMI > 35) or severe obesity (BMI > 40), aged 18 years or older, seeking bariatric surgery at JAAH, Kuwait and enrolled for MDT screening.

##### Eligible procedures

Roux-en-Y, laparoscopic sleeve gastrectomy, laparoscopic gastric band procedures.

##### Exclusion criteria

Patients seeking bariatric surgery at private or other governmental hospitals, as well as those who have previously undergone bariatric surgery.

### Ethical considerations and data handling

Ethical approvalwas obtained from the Ministry of Health, Kuwait Ethical Committee (project no. 2323/2023). All methods were performed in accordance with the relevant guidelines and regulations. Informed consent was obtained from all patients, and data collection was standardized via the Health Information System at JAAH. Data was coded and entered into a spreadsheet, with each patient assigned a unique identifier for anonymity, maintaining privacy and confidentiality. All collected data was stored in a secured and encrypted database, with access restricted to authorized investigators. The study is registered on ClinicalTrials.gov (NCT06480058).

### Handling missing data

Participants who did not meet the inclusion criteria or had missing data for exposure and outcome variables were excluded, resulting in a final analysis sample of 275 participants (detailed in supplementary methods).

### Primary outcome

#### Eating disorder risk screening

The SCOFF questionnaire was self-administered by participants attending their first preoperative appointment. The SCOFF questionnaire is a 5-question test to assess the possible presence of an ED. The S in SCOFF stands for “sick” (to vomit). The O stands for “one stone” of weight (i.e., 6.35 kg). The letters C, F and F stand for “control”, “fat” and “food” respectively. The SCOFF is not used for diagnostic purposes, a score of 2 or more positive answers raises the index of suspicion of EDs^12,13^. The SCOFF has been validated in Arab populations^14^.

### Variables tested as determinants of eating disorder risk

#### Individual characteristics

Demographic data, social risk factors for obesity (e.g., socioeconomic status, family history of individuals with obesity or related co-morbidities), and lifestyle risk factors (e.g., smoking) were obtained using self-administered questionnaires and hospital medical records. Participants’ clinical medical records were used to record the degree of obesity related co-morbidities. Biochemical measures (HbA1c, total cholesterol and triglycerides) were recorded. Anthropometric measures were measured using standard protocols by a trained nurse during preoperative appointments. Body Mass Index (BMI) was calculated using WHO cut-offs to classify obesity [obesity class I (BMI 30–34.9 kg/m^2^), obesity class II (35–39.9 kg/m^2^) and obesity class III (BMI > 40 kg/m^2^)].

#### Depression and anxiety

Psychological screening and assessment for anxiety and depression was carried out by a clinical psychologist using a validated questionnaire. The Beck Depression inventory (BDI) which includes a 21-item multiple choice self-report inventory was used to measure depressive symptoms within the last week. Each question includes a set of 4 possible choices of how a participant may feel. Choices are scored from 0 to 3, increasing in intensity. The cut-offs to indicate depression include 0–9 minimal depression, 10–18 mild depression, 19–29 moderate depression, 30–63 severe depression^15^. The BDI has been validated in Arab populations with Cronbach’s alpha ranging from 0.82 to 0.93^16^. The Kuwait University Anxiety Scale (KUAS) was used to measure level of anxiety. The 20-item scale captures cognitive/affective anxiety (9 items), subjective anxiety or nervousness (7 items), and somatic anxiety (4 items). Responses use a 4-point Likert scale ranging from 1 = rare, 2 = sometimes, 3 = often, 4 = always to measure the level of anxiety. The cut-offs to indicate anxiety include 0–21 normal, 22–42 mild anxiety, 43–63 moderate anxiety, 64–84 severe anxiety^16^. Reliability assessment of the KUAS in this study found Cronbach’s alpha to be at 0.72.

### Analyses

All statistical analyses were performed using SPSS (version 29). Descriptive statistics were carried out to summarize the demographic and investigated psychological, anthropometric and biochemical characteristics of participants. Means and standard deviations will be used for continuous variables and frequencies and percentages were used for categorical data. We calculated total SCOFF score and categorized participants into two groups for ED risk using the SCOFF cut-off (2 or more indicating ED risk). We present characteristics of the study population overall and stratified by SCOFF cut-off.

Logistic regression analyses were performed to investigate associations between demographic and investigated psychological, anthropometric and biochemical characteristics with eating disorders (SCOFF > 2). Statistically significant variables in the unadjusted simple logistic regression analyses were included in the adjusted logistic regression model. An Odds Ratio (OR) with 95% Confidence Intervals (CI) were calculated. Linear regression was used to investigate associations between SCOFF score and demographic, psychological, anthropometric and biochemical variables. Variables that reached significance in the univariate analysis were included in the multivariate analysis as confounders. A beta coefficient with 95% confidence interval (CI) was calculated to identify variables associated with SCOFF score. Statistical significance was set at *p* < 0.05 for all analyses.

## Results

### Patient characteristics

We analyzed data of 275 participants (Table 1): Mean age was 38.5 years (SD = 11.8), 62.5% (*n* = 172) were female, mean body mass index (BMI) was 42.3 kg/m^2^ (SD = 6.8), and 20.7% (*n* = 57) were using nicotine. Looking at overall metabolic comorbidities, for obesity classification (class I: BMI of 30–34.9 kg/m^2^; class II: BMI of 35–39.9 kg/m^2^; class III: BMI of ≥ 40 kg/m^2^), 8% (*n* = 22) of participants were living with class I obesity, 34.9% (*n* = 96) with class II obesity and 57.1% (*n* = 157) with class III obesity. In terms of diabetes, 64.4% (*n* = 177) did not have diabetes, 20.4% (*n* = 56) had prediabetes and 15.3% (*n* = 42) had type 2 diabetes (T2D). Lastly, 19.6% (*n* = 54) were hypertensive. Mean HbA1c was 5.8% (SD = 1.3), total cholesterol and triglycerides were respectively 5.3 mmol/L (SD = 3.4) and 1.4 mmol/L (SD = 0.9).

**Table 1:** Characteristics of study population.

	All		SCOFF < 2		SCOFF ≥ 2		*p* value^a^
	*n* = 275		*n* = 94		*n* = 181		
Demographic, social, lifestyle, obesity							
Age, years	38.5	± 11.8	41.6	± 11.7	36.9	± 11.6	< **0.01**
Gender ^b^							0.835
Females, n (%)	172	(62.5)	58	(61.7)	114	(63.0)	
Males, n (%)	103	(37.5)	36	(38.3)	67	(37.0)	
Weight, kgs	117.2	± 24.9	117.3	± 25.7	117.1	± 24.5	0.935
Height, cm	165.9	± 9.3	165.8	± 9.7	165.9	± 9.2	0.947
BMI, kg/m^2^	42.3	± 6.8	42.5	± 7.0	42.2	± 6.7	0.757
Smoking ^b^							0.430
No, n (%)	218	(79.3)	72	(76.6)	146	(80.7)	
Yes, n (%)	57	(20.7)	22	(23.4)	35	(19.3)	
Obesity classification ^b^							0.125
Obesity class I, n (%)	22	(8.0)	11	(11.7)	11	(6.1)	
Obesity class II, n (%)	96	(34.9)	27	(28.7)	69	(38.1)	
Obesity class III, n (%)	157	(57.1)	56	(59.6)	101	(55.8)	
Clinical and biochemical factors							
Diabetes ^b^							< **0.05**
No, n (%)	233	(84.7)	73	(77.7)	160	(88.4)	
Yes, n (%)	42	(15.3)	21	(22.3)	21	(11.6)	
Diabetes category ^b^							0.057
Normal, n (%)	177	(64.4)	54	(57.4)	123	(68.0)	
Prediabetes, n (%)	56	(20.4)	19	(20.2)	37	(20.4)	
Type 2 Diabetes, n (%)	42	(15.3)	21	(22.3)	21	(11.6)	
Hypertension ^b^							0.146
No, n (%)	221	(80.4)	71	(75.5)	150	(82.9)	
Yes, n (%)	54	(19.6)	23	(24.5)	31	(17.1)	
Biochemical data							
HbA1c, %	5.8	± 1.3	6.1	± 1.5	5.7	± 1.1	< **0.05**
Total cholesterol, mmol/L	5.3	± 3.4	5.7	± 5.6	5.0	± 1.0	0.126
Triglycerides, mmol/L	1.4	± 0.9	1.5	± 1.1	1.4	± 0.8	0.734
Psychological data							
Anxiety – KUAS score	33.8	± 11.8	31.5	± 7.5	35.0	± 10.0	< **0.01**
Anxiety category I^b^							< **0.01**
Normal, n (%)	3	(1.1)	3	(3.2)	0	0	
Mild anxiety, n (%)	232	(84.4)	84	(89.4)	148	(81.8)	
Moderate anxiety, n (%)	36	(13.1)	7	(7.4)	29	(16.0)	
Severe anxiety, n (%)	4	(1.5)	0	0	4	(2.2)	
Anxiety category II^b^							< **0.05**
Normal/mild n (%)	235	(85.5)	87	(92.6)	148	(81.8)	
Moderate/severe anxiety, n (%)	40	(14.5)	7	(7.4)	33	(18.2)	
Depression - BDI score	12.0	± 7.7	9.7	± 5.8	13.2	± 8.4	< **0.001**
Depression category I^b^							< **0.01**
Minimal depression, n (%)	125	(45.5)	53	(56.4)	72	(39.8)	
Mild depression, n (%)	112	(40.7)	37	(39.4)	75	(41.4)	
Moderate depression, n (%)	27	(9.8)	3	(3.2)	24	(13.3)	
Severe depression, n (%)	11	(4.0)	1	(1.1)	10	(5.5)	
Depression category I^b^							< **0.001**
Minimal/mild depression, n (%)	237	(86.2)	90	(95.7)	147	(81.2)	
Moderate/severe depression, n (%)	38	(13.8)	4	(4.3)	34	(18.8)	
Eating Disorder - SCOFF score	2.03	± 1.12	0.76	± 0.43	2.69	± 0.75	–
Eating Disorder category							
SCOFF score < 2, n (%)	94	(34.2)		–	–	–	–
SCOFF score ≥ 2, n (%)	181	(65.8)		–	–	–	–

Data presented as sample size and percentages for categorical variables n (%) and mean ± standard deviation for continuous variables.

^a^Comparison between groups using independent t-test, except ^b^Comparison between dichotomous variables using chi-squared test.

Statistical significance set at *p* < 0.05.

Obesity class I (30–34.9 kg/m^2^). Obesity class II (35–39.9 kg/m^2^). Obesity class III (> 40 kg/m^2^).

*KUAS* Kuwait University Anxiety Scale, *BDI* beck depression inventory.

Significant values are in bold.

Psychological screening included eating disorder risk (SCOFF)^12^, anxiety (KUAS)^16^ and depression (BDI)^15^. Mean SCOFF score was 2.03 (SD = 1.12), approximately two thirds (65.8%; *n* = 181) had a SCOFF score of 2 or more indicating a likely ED. The mean KUAS score was 33.8 (SD = 11.8). Mild anxiety was the most common category, reported by 84.4% (*n* = 232) of participants. Moderate and severe anxiety affected 13.1% (*n* = 36) and 1.5% (*n* = 4), respectively. Mean depression score was 12.0 (SD = 7.7), with minimal or mild depression present in 45.5% (*n* = 125) and 40.7% (*n* = 112) of participants, respectively; and moderate or severe depression present in 9.8% (*n* = 27) and 4% (*n* = 11) of participants, respectively.

### Comparison of ED risk (SCOFF) categories

Based on SCOFF score cut-offs of < 2 or ≥ 2, we analyzed characteristics of participants with or without a likely ED (Table 1).

#### Age, gender, BMI and smoking

Compared to participants with no indication of an ED (SCOFF < 2), those with a likely ED (SCOFF ≥ 2) were of younger age (36.9 SD = 11.6 vs. 41.6 SD = 11.7 years; *p* < 0.01). BMI, gender, smoking status and class of obesity were not different between SCOFF categories.

#### Diabetes, hypertension and biochemistry

Patients with T2D were more represented in the SCOFF < 2 group than the SCOFF ≥ 2 group (22.3% vs. 11.6%; *p* < 0.05). When those at risk and with prediabetes, are also considered, diabetes status remained near-significantly different between SCOFF categories (*p* = 0.057). Participants without diabetes nor prediabetes were more represented in the SCOFF < 2 group than the SCOFF ≥ 2 group (57.4% vs. 68%). Prediabetes status was not different between groups. HbA1c was higher in the SCOFF < 2 group than the SCOFF ≥ 2 group (6.1 SD = 1.5 vs. 5.7 SD = 1.1; *p* < 0.05).

Hypertension was not different between SCOFF categories, as well as total cholesterol and triglycerides.

#### Anxiety and depression

The SCOFF < 2 group had a lower mean anxiety score than the SCOFF ≥ 2 group (31.5 SD = 7.5 vs. 35.0 SD = 10; *p* < 0.01). In anxiety category I, participants with mild anxiety predominated in both comparable groups, although those in the SCOFF < 2 group were more likely to have mild anxiety (*p* < 0.01). In anxiety category II, participants in the SCOFF ≥ 2 group were more likely to experience moderate and/or severe anxiety (*p* < 0.05).

Mean depression score was lower in the SCOFF < 2 group than SCOFF ≥ 2 group (9.7 SD = 5.8 vs. 13.2 SD = 8.4; *p* < 0.001). Grouping scores into categories: depression category I (minimal, mild, moderate or severe; *p* < 0.01) and depression category II (minimal/mild or moderate/severe; *p* < 0.001), participants with minimal depression and minimal/mild depression were more common in the SCOFF < 2 group than SCOFF ≥ 2 group (minimal: 56.4% vs. 39.8%; minimal/mild: 95.7% vs. 81.2%), and moderate and/or severe depression were less common in the SCOFF < 2 group than SCOFF ≥ 2 group (grouped categories: 4.3% vs. 18.8%).

### Univariate linear regression

Considering SCOFF score as a continuous variable with increasing ED risk, we carried out univariate linear regression analysis (Table 2).

**Table 2:** Univariate and multivariate regression.

	Unadjusted			Adjusted		
	β	(95% CI)	*p*	β	(95% CI)	*p*
Age, years	− 0.01	(− 0.02 to 0.00)	0.066	− 0.01	(− 0.02 to 0.01)	0.346
Gender: Male	− 0.10	(− 0.29 to 0.27)	0.945	0.16	(− 0.12 to 0.43)	0.236
Weight, kgs	− 0.002	(− 0.01- 0.00)	0.483	0.002	(− 0.00 to 0.01)	0.515
Height, cm	0.001	(− 0.01 to 0.02)	0.848	0.01	(− 0.01 to 0.02)	0.358
BMI, kg/m^2^	− 0.01	(− 0.03 to 0.01)	0.271	0.001	(− 0.02 to 0.02)	0.937
Smoking: Smoker	− 0.08	(− 0.41 to 0.26)	0.650	− 0.04	(− 0.36 to 0.28)	0.809
Obesity classification						
Obesity class I, n (%)	− 0.32	(− 0.82 to 0.17)	0.197	− 0.18	(− 0.67 to 0.30)	0.458
Obesity class II, n (%)	0.33	(0.05 to 0.61)	< **0.05**	0.34	(0.73 to 0.61)	< **0.05**
Obesity class III, n (%)	− 0.21	(− 0.49 to 0.06)	0.131	0.18	(− 0.30 to 0.67)	0.458
Diabetes category						
Normal, n (%)	0.14	(− 0.15 to 0.41)	0.344	− 0.24	(− 0.59 to 0.12)	0.197
Prediabetes, n (%)	0.15	(− 0.19 to 4.80)	0.384	0.24	(− 0.12 to 0.59)	0.197
Type 2 Diabetes, n (%)	− 0.42	(− 0.79 to − 0.05)	< **0.05**	− 0.24	(− 0.85 to 0.37)	0.436
Hypertension	− 0.40	(− 0.74 to − 0.07)	< **0.05**	− 0.36	(− 0.69 to − 0.61)	< **0.05**
Biochemical data						
HbA1c, %	− 0.11	(− 0.21 to − 0.00)	< **0.05**	− 0.04	(− 0.21 to 0.13)	0.642
Total cholesterol, mmol/L	− 0.02	(− 0.06 to 0.02)	0.406	− 0.03	(− 0.07 to 0.01)	0.137
Triglycerides, mmol/L	− 0.001	(− 0.15 to 0.15)	0.989	0.05	(− 0.09 to 0.19)	0.495
Anxiety - KUAS score	0.02	(0.01 to 0.03)	< **0.01**	0.01	(− 0.01 to 0.02)	0.581
Depression - BDI score	0.03	(0.02 to 0.05)	< **0.001**	0.03	(0.01 to 0.05)	< **0.01**

Linear regression adjusted for Obesity Class II, Depression score, Anxiety score, HbA1c, T2DM category, Hypertension.

Statistical significance set at *p* < 0.05.

*KUAS* Kuwait University Anxiety Scale, *BDI* beck depression inventory.

Significant values are in bold.

#### Age, gender, BMI and smoking

Age had a near-significant negative association with SCOFF score (β = − 0.01, 95% CI − 0.02 to 0.00, *p* = 0.066). Gender, smoking status and BMI were not associated with SCOFF score. Class II obesity was positively associated with SCOFF score (β = 0.33, 95% CI 0.05–0.61, *p* < 0.05).

#### Diabetes, hypertension and biochemistry

T2D was negatively associated with SCOFF score (β = − 0.42, 95% CI − 0.79 to − 0.05, *p* < 0.05). Hypertension was negatively associated with SCOFF score (β = − 0.40, 95% CI − 0.74 to − 0.07, *p* < 0.05). HbA1c was negatively associated with SCOFF score (β = − 0.11, 95% CI − 0.21 to − 0.00, *p* < 0.05). Other variables (normal or prediabetes status, total cholesterol, triglycerides) were not associated with SCOFF score.

#### Anxiety and depression

Anxiety and depression scores were positively associated with SCOFF scores (respectively β = 0.02, 95% CI 0.01–0.03, *p* < 0.01 and β = 0.03, 95% CI 0.02–0.05, *p* < 0.001). Moderate/severe anxiety was also associated with SCOFF score (β = 0.50, 95% CI 0.121–0.873, *p* < 0.05).

### Multivariate linear regression

Following adjustment for class II obesity, anxiety score and depression score, HbA1c, and T2D in the multivariate linear regression (Table 2).

#### Age, gender, BMI and smoking

Class II obesity remained positively associated with SCOFF (β = 0.34, 95% CI 0.73– 0.61, *p* < 0.05).

#### Diabetes, hypertension and biochemistry

Hypertension remained negatively associated with SCOFF score (β = − 0.36, 95% CI − 0.69 to − 0.61, *p* < 0.05). Other variables in this category (normal or prediabetes status, total cholesterol, triglycerides and HbA1c) were not associated with SCOFF score following adjustment.

#### Anxiety and depression

Depression scores were positively associated with SCOFF scores (β = 0.03, 95% CI 0.01–0.05, *p* < 0.01). Anxiety scores were not associated with SCOFF score following adjustment.

### Univariate and multivariate logistic regression

Associations between predictors and SCOFF score ≥ 2 were examined by logistic regressions, univariate (Table 3) or multivariate after adjustment for age, obesity class II, anxiety score, depression score, HbA1c, diabetes category (Table 4).

**Table 3:** Unadjusted (simple) logistic regression to investigate predictors of eating disorder risk (SCOFF^3^ 2).

	OR (95% CI)	*P* value
Age, years	0.97 (0.95–1.00)	< **0.01**
Gender		
Females, n (%)	Ref	
Males, n (%)	0.95 (0.57–1.58)	0.835
Weight, kgs	1.00 (0.99–1.01)	0.935
Height, cm	1.00 (0.97–1.03)	0.947
BMI, kg/m^2^	0.99 (0.96–1.03)	0.756
Smoking		
No, n (%)	Ref	
Yes, n (%)	0.79 (0.43–1.43)	0.431
Obesity classification		
Obesity class I, n (%)	Ref	
Obesity class II, n (%)	2.56 (0.99–6.59)	0.052
Obesity class III, n (%)	1.80 (0.74–4.42)	0.198
Diabetes category		
Normal, n (%)	Ref	
Prediabetes, n (%)	0.86 (0.45–1.62)	0.631
Type 2 Diabetes, n (%)	0.44 (0.22–0.87)	< **0.05**
Hypertension		
No, n (%)	Ref	
Yes, n (%)	0.64 (0.35–1.17)	0.148
Biochemical data		
HbA1c, %	0.79 (0.65–0.96)	< **0.05**
Total cholesterol, mmol/L	0.90 (0.73–1.12)	0.361
Triglycerides, mmol/L	0.96 (0.73–1.25)	0.733
Psychological data		
Anxiety – KUAS score	1.05 (1.01–1.08)	< **0.01**
Depression – BDI score	1.07 (1.03–1.12)	< **0.001**

* KUAS* Kuwait University Anxiety Scale, *BDI* beck depression inventory. *OR* odds ratio, *CI* confidence interval, *Ref* reference group.

Obesity class I (30–34.9 kg/m^2^). Obesity class II (35–39.9 kg/m^2^). Obesity class III (> 40 kg/m^2^).

Statistical significance set at *p* < 0.05.

Significant values are in bold.

**Table 4:** Adjusted logistic regression to investigate predictors of eating disorder risk (SCOFF^3^ 2).

	OR (95% CI)	*P* value
Age, years	0.96 (0.94–0.99)	< **0.01**
Gender		
Females, n (%)	Ref	
Males, n (%)	1.08 (0.61–1.92)	0.801
Weight, kgs	1.00 (0.98–1.01)	0.998
Height, cm	1.00 (0.97–1.03)	0.875
BMI, kg/m^2^	0.98 (0.93–1.04)	0.514
Smoking		
No, n (%)	Ref	
Yes, n (%)	0.68 (0.35–1.32)	0.255
Obesity classification		
Obesity class I, n (%)	Ref	
Obesity class II, n (%)	2.73 (1.02–7.35)	< **0.05**
Obesity class III, n (%)	1.65 (0.64–4.21)	0.299
Diabetes category		
Normal, n (%)	Ref	
Prediabetes, n (%)	1.33 (0.63–2.82)	0.454
Type 2 Diabetes, n (%)	1.17 (0.30–4.57)	0.818
Hypertension		
No, n (%)	Ref	
Yes, n (%)	0.87 (0.42–1.80)	0.706
Biochemical data		
HbA1c, %	0.80 (0.56–1.14)	0.219
Total cholesterol, mmol/L	0.87 (0.67–1.12)	0.300
Triglycerides, mmol/L	1.07 (0.79–1.43)	0.678
Psychological data		
Anxiety – KUAS score	1.02 (0.98–1.06)	0.326
Depression – BDI score	1.07 (1.02–1.13)	< **0.01**

Logistic regression adjusted for age, obesity class II, anxiety score, depression score, HbA1c, diabetes category.

*KUAS* Kuwait University Anxiety Scale, *BDI* beck depression inventory. *OR* odds ratio, *CI* confidence interval, *Ref* Reference group.

Obesity class I (30–34.9 kg/m^2^). Obesity class II (35–39.9 kg/m^2^). Obesity class III (> 40 kg/m^2^).

Statistical significance set at *p* < 0.05.

Significant values are in bold.

#### Age, gender, BMI and smoking

Before adjustment, for every year increase in age, the associated odds in the SCOFF ≥ 2 group decreased by 3% (OR = 0.97, 95% CI 0.95 to 1.00; *p* < 0.01). In the obesity classification variable, patients living with class II obesity were more than double in the SCOFF ≥ 2 group, compared to those with class I obesity, and this was near significant (OR = 2.56, 95% CI 0.99 to 6.95; *p* = 0.052). Gender, smoking status and BMI were not associated with a SCOFF score ≥ 2. After adjustment, age remained significant (OR = 0.96, 95% CI 0.94 to 0.99; *p* < 0.01), and class II obesity remained significant (OR = 2.73, 95% CI 1.02 to 7.35; *p* < 0.05).

#### Diabetes, hypertension and biochemistry

Having T2D decreased the odds of having a SCOFF score ≥ 2 (OR = 0.44, 95% CI 0.22 to 0.87; *p* < 0.05). Lower HbA1c also decreased the odds of having a SCOFF score ≥ 2 (OR = 0.79, 95% CI 0.65 to 0.96; *p* < 0.05). Hypertension, total cholesterol and triglycerides were not associated with SCOFF category. After adjustment, none of these variables were associated with SCOFF category.

#### Anxiety and depression

Before adjustment, both anxiety and depression scores were positively associated with a SCOFF score ≥ 2 (anxiety score: OR = 1.05, 95% CI 1.01 to 1.08; *p* < 0.01; depression score: OR = 1.07, 95% CI 1.03 to 1.12; *p* < 0.001). After adjustment, only depression score remained positively associated with a SCOFF score ≥ 2 (OR = 1.07, 95% CI 1.02 to 1.13; *p* < 0.01). No significant interactions between depression (BDI) score, BMI, gender and age were identified.

## Discussion

Bariatric surgery is the most effective curative solution for obesity, also reducing risk of developing comorbidities. However, EDs negatively impact perioperative and long-term outcomes because of poor adherence to postoperative dietary guidelines, altered metabolic responses and disrupted hormonal signaling^17,18^. Identifying individuals at risk of an ED, by improved risk profiling, will help deliver targeted support to improve outcomes.

The current cross-sectional analysis addressed preintervention prevalence and correlates of ED risk among candidates for bariatric surgery in the BariPredict cohort (NCT06480058). BariPredict is a prospective study that evaluates psychological and physiological factors that influence perioperative and long-term outcomes of bariatric surgery. We found that 65.8% of patients with obesity were at high risk of an ED. The factors associated with ED risk were class II obesity, age and depression score. We performed binary logistic regression treating SCOFF score as a categorical variable, applying the recognized ED risk cut-off of ≥ 2 to define categories. We then validated the associations with SCOFF score as a continuous variable.

### ED prevalence in pre-intervention bariatric surgery candidates

We report an ED risk prevalence of 65.8% in our cohort of obese patients seeking bariatric surgery. This is higher than previous estimates based on the SCOFF questionnaire, reporting 17–32% and 47% in similarly sized European cohorts with obesity, without weight loss intervention^19,20^. The ED risk prevalence we observe also exceeds that reported in persons with obesity from the same geographic region (Saudi Arabia), estimated at 38.3% based on SCOFF screening^21^. Among postoperative patients, ED prevalence was estimated at 7.8% in a systematic review and metanalysis of 7 studies^8^. Reasons for discrepancies in reported prevalences may be related to, demographic, ethnic or geographic differences between cohorts, factors which were not systemically reported. However, the active seeking of bariatric surgery intervention may have resulted from a level of disordered eating which could not be resolved, allowing for weight management by other interventions. Our finding indicates that persons with obesity seeking bariatric surgery may be considered a high-risk group for EDs.

### ED risk is positively associated with class II obesity

Class II obesity is positively associated with SCOFF score, whereas neither class III obesity nor BMI are associated with SCOFF score. This suggests that discrete obesity classifications may capture specific risk profiles more effectively than a continuous BMI measure^22,23^. Class II obesity may represent a critical threshold where individuals experience societal pressure, self-stigma, and related psychological stressors, leading to increased disordered eating behaviors. Additionally, lack of association between ED and BMI indicates the relationship may be non-linear, or that ED risk reaches a plateau above a given BMI point.

These findings underscore the importance of considering obesity classifications in prioritizing psychological assessments and support. Since other reports only partly support the association we found^24,25^, future work will seek to validate it in a larger sample. Additionally, to avoid potential selection bias, we may consider constituting a nested cohort of patients that are eligible for, but choose to opt out of, surgical intervention.

### ED risk is negatively associated with age

We find that younger adults are at higher risk of EDs, this was significant when comparing SCOFF categories, and by logistic regression adjusted for age, class II obesity, anxiety score, depression score, HbA1c, diabetes category. This aligns with existing literature on the prevalence and onset of EDs^26,27^. While our cohort is composed of adults (mean age 38.5 years +/- 11.8), younger individuals within this group will face unique challenges that can contribute to ED risk. Indeed, the mean age of patients with a SCOFF ≥ 2 was 36.9 years, compared to those with a SCOFF < 2 with a mean of 41.6 years. These challenges include body image concerns, sociocultural influences, stress related to life transitions, and less well-developed resilience^28^. Younger adults are also more susceptible to mental health issues such as anxiety and depression, which are linked to EDs^29^.

From this finding, younger adults may be considered a higher risk group for psychological assessment and support prior to bariatric surgery. Deciphering specific vulnerabilities could improve outcomes.

### ED risk is positively associated with depression

The positive association between depression and SCOFF scores in our study confirms a known connection between depression and EDs, consistent with existing literature demonstrating co-occurrence is due to shared socio-psycho-biological pathways^30^. Depressed individuals may engage in emotional eating, forming part of disordered eating patterns^31^. Furthermore, depression can impair motivation and self-regulation, resulting in poor dietary choices and irregular eating behaviors^32^. Neurochemical imbalances that are common in both depression and EDs could further exacerbate this relationship^33^.

Here, patients with depression are also high risk for EDs. If the condition was previously diagnosed, patients with depression are candidates for close monitoring for EDs. Importantly, if the depression or the ED are not both addressed, they may lessen the intervention’s impact, or lead to undesirable outcomes.

### Potential genetic drivers of disordered eating

Although our study did not include genetic testing, certain forms of obesity do have a strong genetic basis—such as monogenic obesity or melanocortin 4 receptor (MC4R) deficiency—that predisposes individuals to binge eating or other forms of disordered eating^34,35^. These genetic factors may also contribute to suboptimal responses to bariatric surgery in some patients, as underlying hyperphagia can persist despite anatomical changes^36,37^. Recognizing patients at high genetic risk—such as those with early-onset obesity, strong familial patterns, or syndromic features—could help clinicians tailor interventions and follow-up strategies. Future work should incorporate genetic screening or targeted evaluations to clarify the impact of monogenic drivers on ED risk and bariatric surgery outcomes.

### Limitations and methodological concerns

Whilst the study used validated tools for psychological assessment, limitations should be acknowledged. Firstly, since SCOFF, KUAS and BDI rely on self-reporting, these may increase self-report/response bias due to social desirability or stigma. Moreover, our cross-sectional design limits inferring causality; and the complexity of relationships between ED and investigated factors may be bidirectional or confounded by factors that were not investigated. Prospective studies will investigate temporal dynamics of these associations and how they influence outcomes.

The SCOFF screening tool does not have the diagnostic accuracy of comprehensive clinical evaluation. Similarly, the BDI captures severity of depressive symptoms, yet is not designed to provide a diagnosis. Results reported from these tools should be taken as risk or severity indicators. As such, although validated for their purpose, they are not appropriate to capture the full complexity of conditions or interactions^38,39^.

The KUAS scale, less widely applied than SCOFF and BDI, has been validated on the background study population, and was evaluated in different ethnic and geographic contexts^16,40–42^. Reliability of the KUAS scale, against the BDI, in our study is as previously reported (Cronbach alpha 0.718)^16^.

Temporal variability should also be considered, such that psychological burdens may fluctuate and may not reflect usual experiences. Investigating the factors that influence ED, anxiety and depression (e.g., life stressors, anticipating surgical intervention) would be valuable.

The small sample size included from the BariPredict cohort also limits the generalizability of our findings and the power per analysis. The post-hoc calculated power for the association of ED with class II obesity is the most reliable at 99%, whereas the associations with age and depression are powered much lower at between 5 and 7%. Another factor that may influence power is the number of analyses, here we compared characteristics of ED risk categories, and carried out linear and logistic regression (univariate and multivariate). Although multiple analyses were carried out to cross-validate findings, this does also increase the chance of type I error per analysis.

Case exclusion due to missing data may also impact statistical outcomes in some circumstances. We carried out additional analyses to address this. Comparing complete cases (*n* = 275) to the screened and enrolled cases that were excluded (*n* = 101), for not meeting study criteria or due to missing data, we found no significant differences in any variables (except for an overweight BMI category being represented among excluded cases) (data not shown). We also tested associations with SCOFF score on all available cases (*n* = 376) and found no significant differences as compared to the complete-case analysis only (data not shown).

## Conclusion

We report prevalence and correlates of ED risk in patients with obesity opting for, but not yet undergone, bariatric surgery. In the BariPredict cohort, the 65.8% prevalence of persons at risk of an ED is higher than expected. By analyzing other available data, an at-risk patient profile is defined by class II obesity, younger age and concurrent depression. The most powerful indicator from these is class II obesity.

Association with depression and age were expected results^24,25^. However, a new finding is the result tied to a specific obesity class, which may indicate a critical window in terms of BMI range that defines a risk profile. Interestingly, we did not report any association with gender, nor with anxiety or metabolic comorbidities.

We will elaborate on these finds in the continuation and follow-up of the BariPredict cohort. While our results indicate which patient profiles should be prioritized for psychological screening and support, future work will investigate temporal dynamics of ED risk and how these impact perioperative and long-term outcomes of bariatric surgery.

## Electronic supplementary material

Below is the link to the electronic supplementary material.

Supplementary Material 1

## Data Availability

The BariPredict study is ongoing and data will be made available upon the study’s completion and upon reasonable request. Contact the first and corresponding authors (Dana AlTarrah, Fawaz Alzaid) of this study for requests related to data.
