# Supplementary material for: Cross-sectional analysis of eating disorder risk and risk correlates in candidates for bariatric surgery from the BariPredict cohort
Source: Sci Rep. 2025 Apr 1;15:11191. doi: 10.1038/s41598-025-95614-6 (PMC11961600; doi:10.1038/s41598-025-95614-6)
Supplement: Supplementary file 1 — Supplementary Material 1 [file 41598_2025_95614_MOESM1_ESM.docx]

**Supplementary material:**

Supplementary Figure S1 Page 2

Supplementary methods :

Pre-participation procedures Page 3

Handling missing data Page 3

**Figure S1**

Complete data for outcome of interest (n=275)

Complete data for exposure variables (n=289)

Missing data in exposure

variables (n=76)

Met exclusion criteria or did not meet inclusion criteria (n=11)

Met inclusion

criteria (n=365)

Enrolled from MDT

screening (n=376)

Missing data in outcome of

interest (n=14)

Included in cross-sectional

analysis (n=275)

**Supplementary Figure 1. Patient enrollment, inclusion and exclusion.** 376 patients were enrolled at MDT screening, 11 met exclusion criteria or did not meet inclusion criteria, 76 had missing data in exposure variables, 14 had missing data in outcome of interest, 275 patients were included in cross-sectional analysis. MDT: Multidisciplinary team.

**Supplementary methods**

**Pre-participation procedures**

Patients seeking bariatric surgery were screened by a MDT. Screening includes multiple steps to assess patients’ suitability and readiness for surgery. The MDT included, a bariatric surgeon, primary care physician, dietitian/nutritionist, psychologist/psychiatrist, nurse coordinator, physiotherapist, and other specialists (endocrinologist, cardiologist, pulmonologist to provide additional evaluations based on comorbidities). Following an initial consultation with the bariatric surgeon, a physical examination is carried out (anthropometry, medical history, laboratory testing/diagnostics, cardiac evaluation, abdominal ultrasound, psychological evaluation, nutritional assessment, medical clearance based on patients’ comorbidities). Following MDT approval, patients are provided with preoperative instructions (preoperative diet, medication adjustments, smoking cessation etc.). Patients are also encouraged to join the educational support group to gain information about the surgery, the recovery process, and post-operative diet progression. Case-by-case guidance is provided to minimize surgical risks and enhance success of surgery.

**Handling missing data**

In this study (n= 376), participants who did not meet the inclusion criteria or had missing data for exposure and outcome variables were excluded, resulting in a final analysis sample of 275 participants. These included missing values in the exposure variables age (n=2), smoking (n=31), depression category (n=1), height (n=1), diabetes category (n=21), hypertension (n=2), ast (n=3), bilirubin (n=1), alt (n=4), creatine (n=1), prothrombintime (n=4), total cholesterol (n=5). Missing values in the outcome variable SCOFF score (n=14). excluded based on predefined inclusion and exclusion criteria age<18 (n=9), BMI<30 (n=2).
